# Genome Sequence of Actinobacteriophage BaronJohn, Isolated on *Microbacterium foliorum* on the College of Coastal Georgia Campus

**DOI:** 10.17912/micropub.biology.002368

**Published:** 2026-10-22

**Authors:** John Barron, John Barton, Brittni Brown, Madeleine Culbreath, Phoebe Gerlyn De Jesus, Elena Doppel, Paige Gailey, Brenda Garcia, Mark Quarterman, Kylie Thomas, Joanna Thomason, Felicia Tran, Kevin Tran, Holly Nance

**Affiliations:** 1 College of Public Health, University of Georgia, Athens, Georgia USA; 2 Natural Sciences, College of Coastal Georgia, Brunswick, Georgia USA

## Abstract

Actinobacteriophage BaronJohn was isolated from soil collected near the Lakeside dormitory building on the campus of the College of Coastal Georgia using *Microbacterium foliorum* NRRL B-24224 as a host. BaronJohn has a genome length and gene content similar to that of phages in cluster EA5. Specifically, BaronJohn encodes a tail assembly chaperone with a -1 translational frameshift, a tRNA-Ala gene, two glycosyltransferases in tandem and a lysin A downstream of the minor tail proteins.

**Figure 1. Phamerator map of BaronJohn’s genome:** Genes are presented as boxes along a genome ruler (in kilobases), with different colors representing different gene families based on sequence similarity. Boxes above the ruler represent genes that are transcribed in the forward direction and genes below the ruler are transcribed in the reverse direction. All predicted gene functions are labeled.

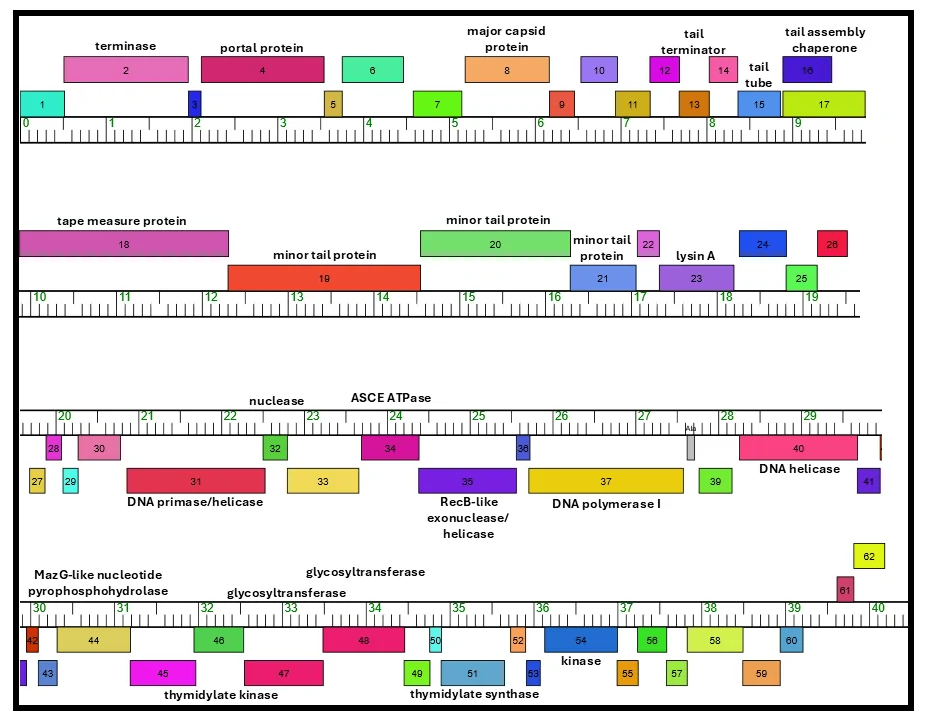



## Description

As the most abundant biological lifeforms on the planet, bacteriophages provide critical roles within microbes and help regulate their populations (Hatfull 2015). Characterizing newly isolated bacteriophages expands not only our knowledge, but it may also reveal novel genes within their genomes that hold biological significance for our species (Bailly-Bechet et al. 2007). This knowledge can provide insight into the ever-expanding host-virus dynamic and theoretically give rise to therapeutic clinical applications, for example to isolate and evaluate bacteria with pathogenic specificity to combat potential diseases (Pope et al. 2015). As antibiotic resistance continues to rise, phage therapy has gained renewed interest as a potential alternative treatment. Due to their high specificity and ability to lyse bacterial cells, bacteriophages may offer a promising solution to multidrug-resistant infections (Zalewska-Piątek 2023).

The *Microbacterium* phage BaronJohn was isolated from a soil sample collected in Brunswick, GA (31°10'56" N, 81° 21' 59" W) using an enriched isolation protocol (Zorawik et al. 2024). The soil sample was incubated in a peptone-yeast-calcium (PYCa) liquid medium at 32°C while shaking for two hours at 360 RPM using a Labnet Orbit™ 300 Digital Multipurpose Shaker. After incubation, the sample was centrifuged at 2,000 x g for 10 minutes and the supernatant was filtered through a 0.22 µm pore size. This supernatant was collected in a vented 50 ml conical tube, seeded with 0.5 ml of *Microbacterium foliorum* , and incubated at 32°C with shaking at 360 RPM for three days. Following incubation, the supernatant was filtered through a 0.22 µm pore size membrane, and serial dilutions (10 ^-1^ to 10 ^-8^ µl) were plated with host *M. foliorum* on PYCa medium with soft agar overlay. After 48 hours of incubation at 32°C, clear, well-defined plaques were observed. BaronJohn was purified through four rounds of plaque assays.

Phage DNA was extracted from high-titer lysate (6 x 10 ^10^ pfu/ml). Briefly, bacterial DNA and RNA in the lysate was first degraded by treatment with DNase I and RNaseA. Nucleases were subsequently inactivated with EDTA, Proteinase K, and SDS , after which phage DNA was extracted using the Promega Wizard DNA Cleanup System. Libraries were prepped using the NEB II Ultra Library kit and sequenced using Illumina NextSeq 1000, producing 2,501,030 single-end 100-bp reads (5077-fold coverage). Raw reads were trimmed with Cutadapt 4.7 (Miller 2011), using the option: –nextseq-trim 30, and filtered with Skewer 0.2.2 (Jiang et al. 2014), using the options: -q 20 -Q 30 -n -l 50, prior to assembly. BaronJohn's genomic termini were assessed using Consed v29 (Gordon and Green 2013) following Russell (2018) and raw reads were assembled using Newbler v2.9 (Miller et al. 2010). The genome of BaronJohn is 40,502 base pairs long and circularly permuted with a G+C content of 63.9%.

Glimmer v3.02 (Delcher et al. 2007) and GeneMark.hmm v3.36 (Besemer and Borodovsky 2005) were used in the initial auto-annotation to identify regions with coding potential. DNA Master v5.23.6 (Pope and Jacobs-Sera 2017) and Starterator v587 (http://phages.wustl.edu/starterator/) were used to review these predictions and revise as needed. BLASTp v2.2.26 (Altschul et al. 1990) with the Actinobacteriophage and NCBI non-redundant databases and PhagesDB (Russell and Hatfull 2016) were used to compare sequences and functions of similar genes. Phamerator, using Actino-draft database v643 (Cresawn et al. 2011) was used to compare gene location, length, and conserved domains among closely related phages. HHpred MPI Bioinformatics Toolkit with databases PDB_mmCIF70_20_Feb, SCOPe70_2.08, Pfam-A_v38.2, NCBI_Conserved_Domains (CD)_v3.19, and UniProt-SwissProt-viral70_3_Nov_2021 was used to analyze protein crystalline structure to predict function (Söding et al. 2005). DeepTMHMM v1.044 (Chen et al. 2003) was used to identify transmembrane proteins. Aragorn v1.2.41 was used to confirm the presence and location of tRNA genes (Laslett and Canback 2004). Default settings were used for all software.

BaronJohn was assigned to subcluster EA5 due to at least 35% shared gene content similarity to phages in the Actinobacteriophage database ( https://phagesDB.org ; Russell and Hatfull 2016; Pope et al. 2017). The G+C content of 63.9% falls within the range (63.5-64.4%) for subcluster EA5 phages. BaronJohnhas a total of 61 putative genes, 24 of which were assigned functions, and one tRNA-Ala gene (see Fig. 1 for details). This is consistent with the 15 other phages in subcluster EA5 to date, whose number of genes range from 57-63 per genome, and 10 of 15 phages which also have a tRNA gene that is either Ala or undetermined, and syntenic (https://phagesDB.org). Five of the genes in BaronJohn were found to contain transmembrane domains, although the function could not be determined. Roughly half of the genes (28) are transcribed in the forward direction, with the other half (33) transcribed from the opposite strand. Like all other EA5 phages, BaronJohn encodes a tail assembly chaperone with a -1 translational frameshift, a MazG-like nucleotide pyrophosphohydrolase, and two glycosyltransferases (all EA5 phages annotated to date have either one or two in tandem). All 16 EA5 phages encode a lysin A (previously called endolysin) downstream of the minor tail proteins, although these genes comprise two different gene families (Phamilies). No genes canonically associated with lysogeny (e.g. integrase, immunity repressor) were identified on this genome, suggesting a virulent (lytic) life cycle.

**Nucleotide Accession Number**

GenBank Accession Number is PZ575424 . The Sequence Read Archive (SRA) number is SRX32199476 .
